# Absolute Quantification of Endogenous Ras Isoform Abundance

**DOI:** 10.1371/journal.pone.0142674

**Published:** 2015-11-11

**Authors:** Craig J. Mageean, John R. Griffiths, Duncan L. Smith, Michael J. Clague, Ian A. Prior

**Affiliations:** 1 Division of Cellular and Molecular Physiology, Institute of Translational Medicine, University of Liverpool, Liverpool, L69 3BX, United Kingdom; 2 Cancer Research UK Manchester Institute, University of Manchester, Manchester, M20 4BX, United Kingdom; Moffitt Cancer Center, UNITED STATES

## Abstract

Ras proteins are important signalling hubs situated near the top of networks controlling cell proliferation, differentiation and survival. Three almost identical isoforms, HRAS, KRAS and NRAS, are ubiquitously expressed yet have differing biological and oncogenic properties. In order to help understand the relative biological contributions of each isoform we have optimised a quantitative proteomics method for accurately measuring Ras isoform protein copy number per cell. The use of isotopic protein standards together with selected reaction monitoring for diagnostic peptides is sensitive, robust and suitable for application to sub-milligram quantities of lysates. We find that in a panel of isogenic SW48 colorectal cancer cells, endogenous Ras proteins are highly abundant with ≥260,000 total Ras protein copies per cell and the rank order of isoform abundance is KRAS>NRAS≥HRAS. A subset of oncogenic KRAS mutants exhibit increased total cellular Ras abundance and altered the ratio of mutant versus wild type KRAS protein. These data and methodology are significant because Ras protein copy number is required to parameterise models of signalling networks and informs interpretation of isoform-specific Ras functional data.

## Introduction

Ras proteins are small monomeric G-proteins that control key signalling pathways emanating from cell surface receptors. Mutations at codons 12, 13 or 61 favour prolonged GTP binding and aberrant activation of the Ras signalling network. These mutations are present in approximately 30% of all tumours screened and oncogenic mutations and/or amplification of members of the Ras pathways are present in almost all cancers [1, 2].

Three Ras genes encode four isoforms (HRAS, KRAS4A, KRAS4B and NRAS) that are ubiquitously expressed and almost identical, but not functionally redundant [3]. KRAS is the most frequently mutated isoform in tumours and this has been proposed to be due to DNA sequence-specific susceptibility to mutation [4] or specific spatio-temporal expression patterns versus the other isoforms [5]. Alternatively, isoform-specific protein functions may mediate the particular oncogenic potency of KRAS [6, 7]. Understanding the absolute abundance of Ras isoforms and their mutated alleles is required for critical assessment of these models. Furthermore, Ras quantification will inform the *in silico* modelling of growth factor receptor-Ras network biology and the proposed compartmentalised organisation of Ras pathways [8–12].

Previous analysis of Ras isoform expression has relied on a variety of semi-quantitative or non-comprehensive approaches. Gene expression analysis enabled qualitative comparison of relative mRNA abundance across tissues but did not allow direct comparison of relative expression between the isoforms [13]. Furthermore, mRNA abundance does not necessarily correlate with protein expression levels [14]. Semi-quantitative assessment of the contribution of each isoform to total Ras protein abundance indicated that KRAS was frequently the most abundant isoform across a panel of cancer cell lines [15]. More precise measurements of protein abundance can be made using quantitative mass spectrometry involving calibrated protein or peptide standards. The high degree of protein sequence homology between the Ras isoforms presents a significant challenge for quantitative proteomics by limiting the availability of proteotypic peptides suitable for Ras isoform quantitation. This means that proteomic studies have typically quantified total Ras abundance rather than measured the individual isoforms. Using isotopic Ras peptide standards, a number of groups have directly measured wild type and mutant Ras protein abundance with results ranging from <100 to >5,000,000 copies of Ras protein per cell [14, 16–19].

The significant divergence in estimates of Ras abundance in pancreatic and colorectal cell lines is likely to be due to the enrichment and relative quantitation strategies used. Each of the targeted approaches used to date have spiked in labelled Ras peptide standards immediately prior to injection of the sample into the mass spectrometer. This means that differences and inefficiencies during sample preparation in antibody-based protein enrichment, Ras proteolysis or peptide gel extraction are not factored into the final quantitation of absolute abundance for each sample [20]. To avoid these problems we have optimised a protein standard absolute quantitation (PSAQ) methodology where full-length isotope-labelled Ras standards are spiked into the initial cell lysate with no antibody enrichment steps required [21, 22]. We applied the methodology to a panel of isogenic cells that are engineered to express common oncogenic variants of KRAS from the endogenous locus. This approach allows accurate measurement of endogenous protein copy number of all of the Ras isoforms and can be applied to any biological samples including those from the clinic.

## Materials and Methods

### Materials

^13^C_6_-^15^N_2_-lysine (+8 Da) and ^13^C_6_-^15^-N_4_-arginine (+10 Da) were obtained from Sigma. His-tagged, wild-type KRAS4B and NRAS plasmids were kindly provided by Ignacio Rubio (Institute of Molecular Cell Biology, University of Jena). Wild-type, full-length human HRAS and KRAS4A sequences were sub-cloned into the pTrcHis A vector (Invitrogen) from plasmids described in [23, 24] and sequence verified.

### Ras standard production

His-tagged wild-type human HRAS, KRAS4A, KRAS4B and NRAS were transformed into AT713 *E*.*coli* that are auxotrophic for lysine and arginine biosynthesis (Yale E.Coli Genetic Stock Centre, New Haven USA). Bacteria were grown in 5 ml LB broth at 37°C for 6 hours, before 100 μl of culture was spiked into 100 ml isotopic M9 minimal media (24 mM Na_2_HPO_4_, 11 mM KH_2_PO_4_, 4.3 mM NaCl, 0.2 mM MgSO_4_.7H_2_O, 0.1 mM CaCl_2_, 0.4% [w/v] glucose, 0.1 mg/ml of each hydrophilic amino acid, 0.2 mg/ml of each hydrophobic amino acid, not including arginine and lysine (pH 7.4)), supplemented with (0.2 mg/ml) heavy arginine and lysine, and incubated overnight at 37°C. The culture was diluted into 1 litre of fresh isotopic M9 minimal media and induced for 3 hours with 1 mM IPTG when an OD_600_ of 0.6 was reached. Ras proteins were initially purified using a PrepEase his-tagged protein purification kit (Affymetrix), before being subject to gel filtration using a Superdex 200 10/30 GL and ÄKTA protein purification system (both GE Healthcare). Ras proteins were filtrated in 20 mM Tris (pH 7.4), 150 mM NaCl, 2 mM DTT and 5 mM MgCl_2_ at a 0.5 ml/min flow rate. Ras proteins were stored in protein LoBind tubes (Eppendorf), pre-treated with 2% [w/v] BSA, at -80°C following flash freezing in liquid nitrogen. Labelling of Ras standards was checked by subjecting the purified Ras proteins to in-gel digestion with trypsin or elastase, before selected reaction monitoring (SRM) targeting light and heavy versions of lysine- and arginine-containing peptides. Concentration of the Ras standards was determined by mixing the heavy Ras proteins with a known amount of an accurately quantified light KRAS4B reference protein, ahead of SDS PAGE and in-gel tryptic digestion. Subsequent SRM analysis enabled accurate quantification of the isotope-labelled Ras standards. The light KRAS4B reference protein was expressed as described above, but gel filtrated in PBS, and concentration measurements were made using an absorbance measurement at 280 nm, with a Nanodrop 1000 (Thermo), and a bicinchoninic assay (BCA, Thermo).

### Cell lines, counting and lysis

Isogenic SW48 cells were obtained from Horizon Discovery. The clones used were heterozygous knock-in (G12A/+) of K-Ras activating mutation KRAS^G12A^ (HD 103–009), heterozygous knock-in (G12C/+) of K-Ras activating mutation KRAS^G12C^ (HD 103–006), heterozygous knock-in (G12D/+) of K-Ras activating mutation KRAS^G12D^ (HD 103–011), heterozygous knock-in (G13D/+) of K-Ras activating mutation KRAS^G13D^ (HD 103–002), heterozygous knock-in (G12R/+) of K-Ras activating mutation KRAS^G12R^ (HD 103–010), heterozygous knock-in (G12S/+) of K-Ras activating mutation KRAS^G12S^ (HD 103–013) and heterozygous knock-in (G12V/+) of K-Ras activating mutation KRAS^G12V^ (HD 103–007). These were referenced to homozygous KRAS^WT^ expressing cells (HD PAR-006), hereafter referred to as Parental cells. All cells were grown in McCoy’s 5A media (Invitrogen), supplemented with 10% [v/v] FBS, at 37°C in 5% [v/v] CO_2_.

Cells were grown to 80% confluency in 100 mm dishes, trypsinised and resuspended in 10 ml warm PBS, before being counted using a Bright-Line haemocytometer (Sigma). 9 ml of suspended cells was pelleted through centrifugation at 200 rcf for 5 min. The supernatant was checked to ensure no cell clumps were present, followed by aspiration of PBS without dislodging the cell pellet. 200 μL of ice-cold RIPA buffer (10 mM Tris (pH 7.5), 150 mM NaCl, 0.1% [w/v] Triton X-100, 0.1% [w/v] SDS, 1% [w/v] sodium deoxycholate, supplemented with protease inhibitors; #P8340, diluted 1:250, Sigma Aldrich) was added to the cell pellet and incubated on ice for 20 mins, with regular agitation. Cell volume was measured using a Hamilton syringe (Hamilton Bonaduz AG), ahead of lysate protein concentration measurement using a BCA assay.

### Mass spectrometry analysis and sample preparation

Ras proteotypic peptides were identified by in-gel digestion of pure Ras protein with trypsin Gold (Promega) or elastase (Calbiochem). Approximately 100 ng of the resultant Ras peptides were separated using a nanoAcquity UPLC system (C18, 5 μm particle size, 180 μm × 20 mm, Symmetry, 2G-V/M trap column and C18, 1.7 μm particle size, 75 μm × 250 mm BEH130 column (Waters, MA, USA)), before ionisation using a nanospray II source (AB SCIEX, CA, USA). In this study, mass spectrometry was performed with a 4000 QTRAP (AB SCIEX, CA, USA) using information-dependent acquisition (IDA), MRM-initiated detection and sequencing (MIDAS^**™**^) or SRM. For detection of proteotypic Ras peptides, IDA was utilised. IDA scans comprised of an enhanced mass spectrometry survey scan (EMS) (400–1,200 Th range at 4,000 Th/s scan rate), followed by an enhanced resolution scan (ER) (250 Th/s of top 5 multiply charged ions) to determine the charge state, with the top 2 ions fragmented with an optimised rolling collision energy. MS/MS spectra were acquired using an enhanced product ion scan (EPI) (100–1100 Th range, 4,000 Th/s scan rate). All data were acquired using the vendor’s software, Analyst (version 1.5, AB SCIEX). Data files from IDA scans (.wiff) were converted into.mgf file format and subjected to a Mascot MS/MS ion search (Matrix Sciences, London, UK).

For cellular Ras quantification, 100 μg of cell lysate, with spiked-in isotope-labelled Ras standards (10 ng of KRAS4B, 5 ng of HRAS and NRAS), was subject to SDS PAGE, followed by excision of a gel band that contained both the heavy Ras standards and endogenous Ras proteins. The gel region of interest was first identified using Western blotting and pre-stained molecular weight markers to define easily identifiable upper and lower limits between which endogenous and spiked in Ras proteins were contained. Typically we excised a region corresponding to 17–31 kDa. Proteins were reduced with 10 mM dithiothreitol and alkylated with 55 mM iodoacetamide. In-gel digestion with trypsin Gold was performed at a 1:25 protease: protein ratio (400 ng trypsin) for 16 hours at 37°C, before peptides were extracted with 100% ACN, reaction stopped by adding formic acid to a final concentration of 0.1% and dried in a vacuum concentrator. Peptides were resuspended in 0.1% [v/v] TFA for LC-MS analysis. Assuming complete digestion and peptide recovery, and considering the size of the excised gel piece, an estimated 1 μg of peptides were loaded and trapped for 10 mins at 3 μl/min flow rate in 99.9% [v/v] water: 0.1% [v/v] acetonitrile (ACN) and formic acid (0.1% [v/v] overall), then resolved using a 60-minute, linear 8–35% [v/v] ACN gradient in 0.1% [v/v] formic acid at a flow rate of 400 nl/min, with column temperature at 60°C, using the UPLC equipment detailed above. Proteotypic Ras peptides were quantified using the peak areas obtained from SRM acquisitions using Skyline (version 2.4) [25]. Transitions employed in this study are detailed in S1 Table. Calculation of Ras molecules per cell was performed by dividing the ng of Ras measured by the averaged number of cells counted, before conversion into moles and multiplication by the Avogadro constant.

For detection of mutant-specific Ras peptides, precursor and product ion *m/z* values for the mutant Ras peptides were calculated using Skyline. An estimated 1 μg of peptides was separated as above, before transitions targeting y9, y8 and y7 ions of the mutant-specific Ras peptides were monitored using a MRM-initiated detection and sequencing (MIDAS)-based protocol [26]. MIDAS scans began with a user-defined SRM stage, before ions exceeding 10,000 cps were fragmented as for the IDA. Acquired spectra were inspected manually. All wiff files can be downloaded from Peptide Atlas: http://www.peptideatlas.org/PASS/PASS00743.

## Results

**Fig 1:**
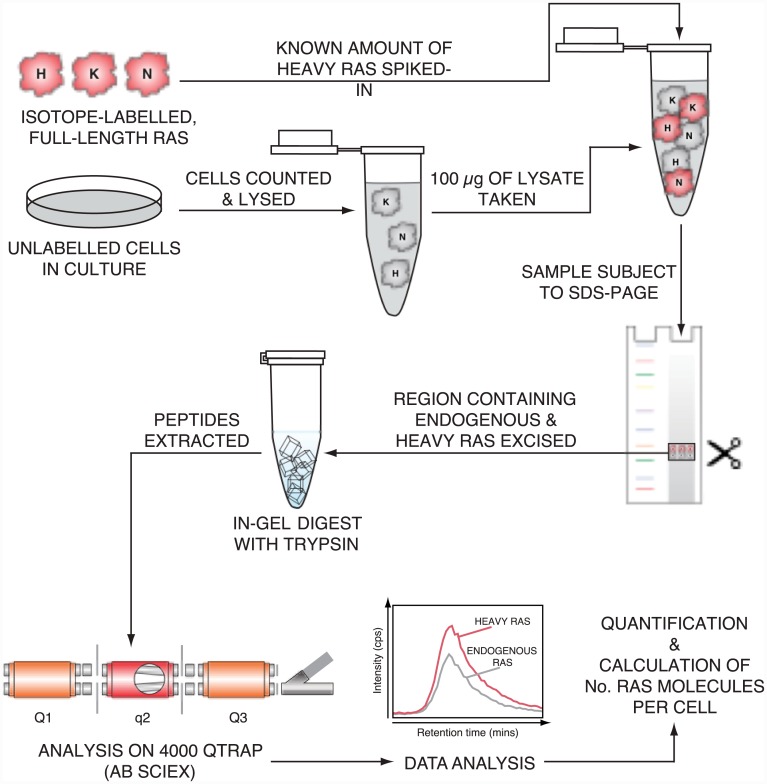
Protein standard absolute quantification for measurement of cellular Ras abundance. Isotopically-labelled Ras proteins are spiked into cell lysates at a known concentration, before fractionation, proteolysis and quantification of pre-defined proteotypic peptides using selected reaction monitoring to allow calculation of Ras isoform abundance.

The experimental strategy for quantifying Ras isoform abundance can be seen in Fig 1. Protein standards incorporating heavy isotopes of Arginine or Lysine are generated, which generate a diagnostic +8Da (Lys) or +10Da (Arg) increase in mass of the peptide enabling differentiation from the light, endogenous peptide and subsequent quantitation through comparison of peak areas. The labelled protein standards are spiked directly into 100 μg of cell lysate representing a known number of cells. No pre-enrichment steps for Ras proteins were necessary; this both simplifies the methodology and removes a major potential source of error in the final estimates associated with non-quantifiable variabilities in immuno-precipitation efficiency. Lysates, with spiked-in heavy Ras standards, were fractionated using SDS PAGE and the area of the gel containing both light and heavy forms of the co-migrating Ras isoforms was excised for subsequent in-gel tryptic digestion. The use of isotope-labelled protein standards spiked in at the start rather than synthetic peptides added at the end of the process ensures that experimental variability in sample processing and variation in trypsin digestion efficiency between peptides and samples does not influence the quantitation of absolute protein abundance [20]. Triple quadrupole-based selected reaction monitoring (SRM) analysis, coupled with liquid chromatography, was utilised to quantify cellular Ras abundance. SRM-based proteomic assays enable precise and sensitive quantification of diagnostic peptides from complex biological backgrounds and require the selection of specific *m/z* settings, for the first and third quadrupole, to select for a particular precursor/ product ion pair known as a transition. All proteotypic Ras peptides were quantified using 5 transitions, with the exception of the KRAS4B specific-peptide and the proline-containing HRAS-specific and shared KRAS and NRAS peptides. Selected peptides and transitions utilised in this study are provided in S1 Table. Transitions were chosen based on intensity, lack of interference from the biological sample and reliability in generating a strong signal during SRM analysis.

**Fig 2:**
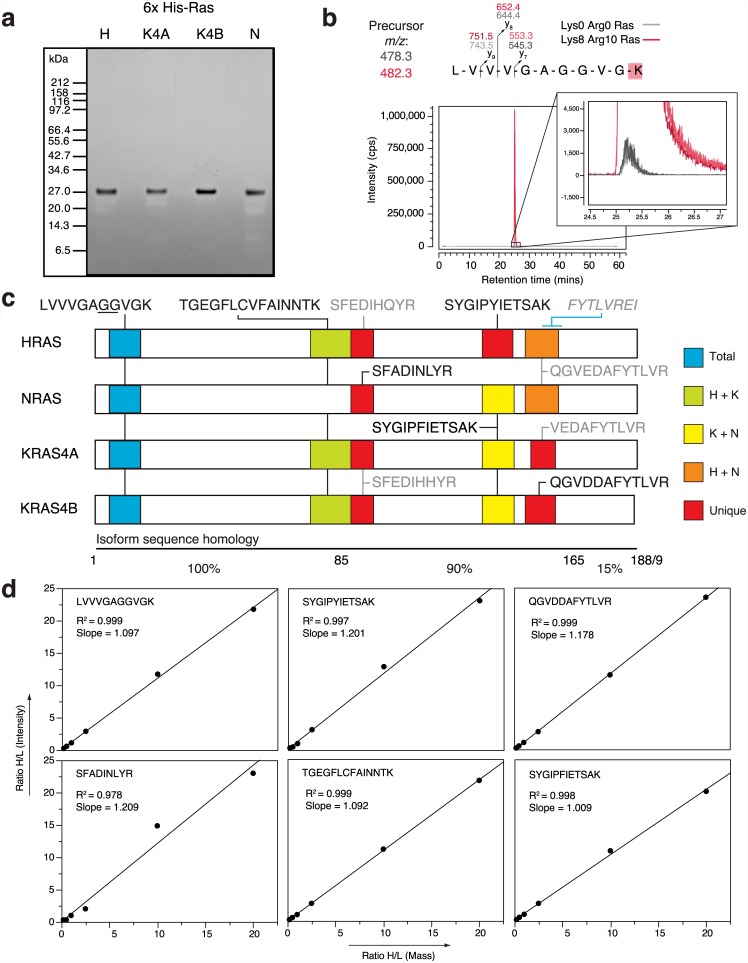
Isotopically-labelled Ras standards and proteotypic peptides used in this study. His-tagged Ras protein standards are isolated to high purity (A) and exhibit >99% incorporation of isotope label (B). (C) Ras peptides used for quantification. Peptides in black were utilised for quantification, while those in grey were identified but were unreliably detected/ undetectable at endogenous levels in SW48 cells. All peptides are tryptic, bar FYTLVREI that is elastase derived. Sites of common Ras oncogenic mutations at codons 12 and 13 are underlined. (D) A linear relationship is observed (R^2^ > 0.97) between the abundance of the Ras peptides injected onto the mass spectrometer and the integrated intensity of transitions. Full-length, light and isotope-labelled Ras proteins were mixed at ratios from 0.2 to 20, before being subject to SDS-PAGE and in-gel tryptic digestion. The peptide mixtures were subject to SRM analysis and data analysed using Skyline.

We generated high purity His-tagged heavy-isotope labelled protein standards for HRAS, NRAS, KRAS4A and KRAS4B (Fig 2A). The protein concentration of each heavy Ras standard was determined by comparison with a known amount of accurately quantified light KRAS4B standard (S1 Fig). Incorporation of isotope-labelled amino acids, ^13^C_6_/^15^N_2_-lysine (+8 Da) or ^13^C_6_/^15^/N_4_-arginine (+10 Da), was >99.8% for every Ras protein (Fig 2B, S2 Fig).

**Fig 3:**
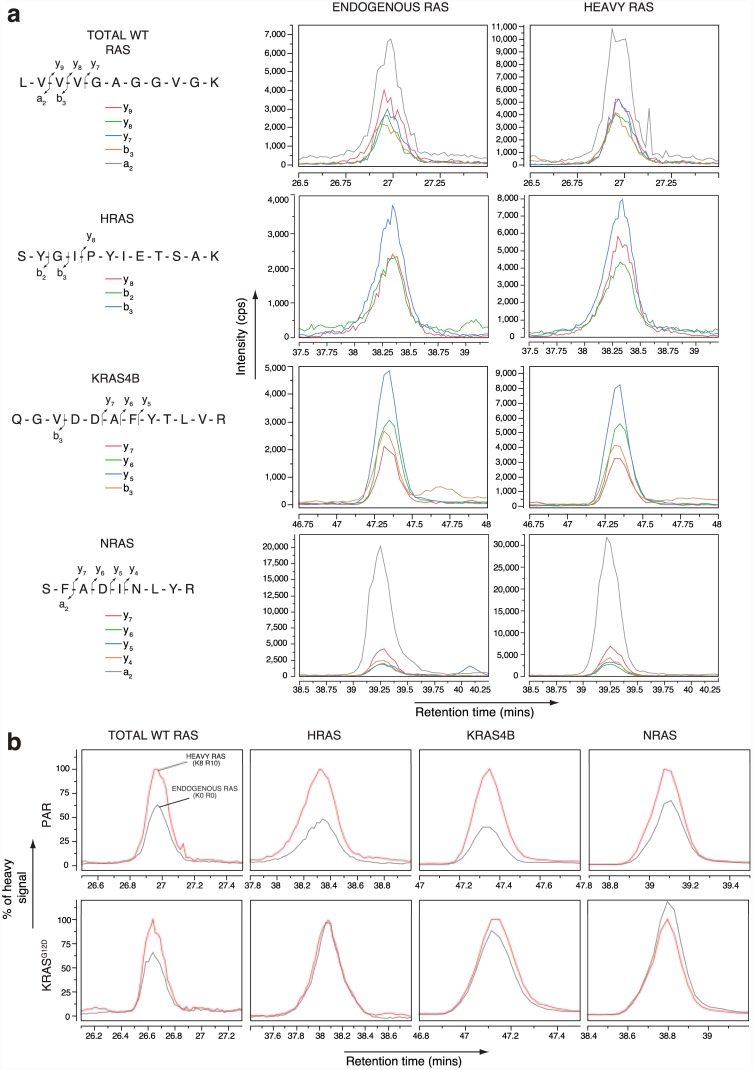
Selected reaction monitoring of proteotypic Ras peptides in SW48 isogenic cell lines. (A) Individual transitions of endogenous (Lys0 Arg0) and heavy (Lys8 Arg10) peptides describing total wild type, HRAS, KRAS4B and NRAS abundance in the Parental (homozygous KRAS^WT^) SW48 cell line. (B) Integrated transitions (normalised to heavy signal) of the proteotypic Ras peptides in Parental (PAR) and heterozygous KRAS G12D knock-in (KRAS^G12D^) SW48 cells. Presented data representative of 3 biological replicates.

The first 85 amino acids of the four Ras isoforms are identical; therefore, peptides from this region provide a read-out for total Ras content of the cell (Fig 2C). Trypsin digestion generates diagnostic peptides for each Ras isoform in the C-terminal half of the proteins (see S5 Fig for MS/MS spectra of each peptide). Empirical analysis revealed a single peptide for each Ras protein that could be reproducibly detected in the mass spectrometer (Fig 2C, red highlighting). Peptides shared between two Ras proteins were included for corroborating quantitation of individual isoforms. All detectable Ras peptides (Fig 2C, black peptides) demonstrated a linear response between peptide abundance and peak areas across a range of ratios between unlabelled Ras and heavy Ras standards (Fig 2D). To verify transitions, we confirmed that the heavy Ras standards and light endogenous cellular peptides co-elute exactly and that transitions preserved their order of intensity (Fig 3A and S3 Fig). When unlabelled and labelled transitions were integrated, the signal intensities for both versions were within a factor of 3 for all peptides tested across all cell lines examined, enabling accurate quantitative analysis (Fig 3B). To assess technical reproducibility, 5 independent acquisitions of a single sample were performed, with each Ras proteotypic peptide showing a variance of ≤ 7.1%. Although we detected the elastase-derived peptide, FYTLVREI, in pure protein digests and utilised this to ensure full heavy arginine incorporation into the Ras protein standards, we could not detect this peptide at endogenous levels using SRM on the 4000 QTRAP. We were also unable to detect the KRAS4A-specific peptide, VEDAFYTLVR, at endogenous levels, most likely because the SW48 cell line expresses little to no mRNA for this KRAS isoform [27].

**Fig 4:**
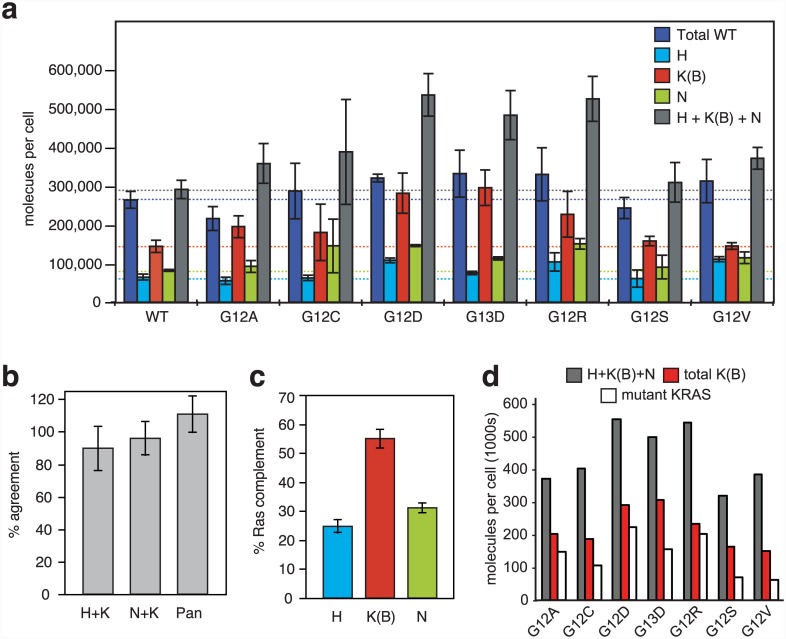
Cellular Ras abundance in isogenic colorectal cell lines. (A) Ras proteins are highly abundant but their levels vary across a panel of isogenic SW48 cells harbouring different oncogenic mutations in KRAS. Horizontal lines indicate the wild-type cell lines to aid comparison. (B) Quantitation of isoform-specific peptides is corroborated by peptides shared between isoforms. Percentage agreement was calculated as ((isoform-specific peptide + isoform-specific peptide) / shared peptide) × 100. Shared peptides were TGEGFLCVFAINNTK (HRAS and KRAS), SYGIPFIETSAK (KRAS and NRAS) and LVVVGAGGVGK (all wild-type isoforms). *n* ≥ 20 for H+K and N+K, *n* = 3 for Pan as only applicable to the wild-type Ras SW48 cell line. Bars represent mean ± SD. No significant difference (p>0.01 paired T-test) is observed between the total estimated molecules per cell of HRAS peptide + KRAS isoform-specific peptidse versus the HRAS/KRAS shared peptide, NRAS + KRAS isoform specific peptides versus KRAS/NRAS shared peptide and H+K+N isoform specific peptides versus the wild type Pan-Ras peptide. (C) KRAS represents >50% of total cellular Ras in the wild-type Ras SW48 cell line. For each graph in each panel, bars represent mean ± SD of ≥3 biological replicates. (D) Number of calculated mutant KRAS molecules changes between cells harbouring different KRAS mutations. Mutant KRAS levels were calculated by subtracting number of wild-type RAS molecules from the sum of H+K(B)+N isoform specific peptides.

To examine cellular Ras abundance we used a panel of isogenic SW48 cells that each harbour distinct oncogenic activating mutations in one allele of the endogenous KRAS gene locus [28]. Parental SW48 cells are wild type for KRAS and contain approximately 290,000 copies of Ras per cell (Fig 4A). To provide independent validation of the accuracy of the quantitation, we compared the summed values from isoform-specific peptides with those peptides present in more than one isoform and observed no significant difference between the pairs of totals (Fig 4B). Our data reveal that all three Ras isoforms are highly expressed in SW48 cells, with KRAS corresponding to 50% of total cellular Ras (Fig 4C). It is likely that KRAS consists almost entirely of KRAS4B in these cells since the KRAS4A-specific peptide could not be detected at endogenous levels.

The isogenic SW48 panel allowed the examination of whether the presence of specific activating mutations of KRAS influences Ras abundance. Importantly, these are measurements of endogenous Ras isoform abundance, rather than based on the use of ectopic expression. Significant variation in Ras isoform abundance was observed between cell lines; although this is most clear for KRAS, the rank order of KRAS>NRAS>HRAS was preserved in every case (Fig 4A). The LVVGAGGVGK peptide is present in all four Ras isoforms however it contains the important G12 and G13 codons that are commonly oncogenically mutated in Ras. Therefore, whilst this peptide accurately reports wild type Ras abundance it will not detect the oncogenically mutated Ras proteins in the SW48 cell panel. Whilst there are no significant changes in wild type Ras protein abundance between the cell lines, an increase in total Ras (HRAS+KRAS4B+NRAS) content was observed in every KRAS mutant cell line compared to Parental wild type cells (dark blue and grey bars, Fig 4A). The most significant increases were seen with the charged amino acid substitutions (G12D, G13D and G12R) that resulted in >65% increases in total Ras levels to >486,000 copies per cell. Notably, the total wild type peptide values do not significantly change between wild type and KRAS mutant cells (dark blue bars, Fig 4A). Therefore, the significant increases in total Ras (HRAS+KRAS4B+NRAS) content most likely represent mutant KRAS that cannot be detected by the pan-Ras wild type peptide.

Since the isogenic cell line panel harbour a range of mutant Ras proteins, this enabled mutant-specific Ras peptides to be targeted using a MIDAS-based approach. Peptides derived from G12D, G12V, G13D, G12A, G12C and G12S mutant Ras were detected (S4 Fig). Although G12D- and G13D-specific peptides share identical precursor masses and a similar product ion series, a diagnostic y4 ion (*m*/z 360.2) was discovered that enables their differentiation.

Intriguingly, the estimated contribution of mutant KRAS to total Ras abundance varies significantly between cell lines (Fig 4D). Whilst mutant KRAS peptides were not directly quantified, we can infer relative abundance by subtracting the wild type pan-Ras total from the (H+KRAS4B+N) isoform-specific peptide total. This reveals that mutant KRAS protein represents approximately 15–40% of total cellular Ras. For the majority of the cell lines, the mutant total represented 50±10% of the KRAS total. However, for G12A, G12D and G12R there appears to be a bias towards mutant allele expression or protein stabilization since >75% of KRAS and ~40% of total cellular Ras is estimated to consist of the mutant protein. Together, these data suggest that oncogenic signalling associated with individual KRAS mutations differentially regulates mutant KRAS dosage.

## Discussion

We have successfully used PSAQ SRM analysis to accurately quantify the endogenous abundance of Ras isoforms in cells. The technique is sensitive and linearly responsive over a wide range of heavy/ light ratios (0.2 to 20) that comfortably encompass the ratios measured in the present study (Fig 2D). A number of previous studies have attempted to quantify Ras abundance with conflicting results. Label free proteomics approaches have quantified 40,000 and 175,000 copies of KRAS per NIH3T3 or HeLa cell respectively [14, 17]. Whilst label free approaches provide an estimate of protein copy number, isotopic labelling strategies allow precise comparisons with a protein or peptide standard. Wang and colleagues used a pan-Ras immuno-precipitation enrichment strategy followed by spiking in AQUA peptide standards to characterise Ras abundance, observing ~0.5–5 pmoles (~10–100 ng) of total Ras per mg of protein in colorectal and pancreatic cell and tissue samples [19]. Given that the immuno-precipitation step was <100% efficient, the relative enrichment could not be accurately quantified, this could have been offset if the isotopic standards had been added before the immuno-precipitation step. Furthermore, the use of isotopic peptide standards does not report sample handling variability or proteolysis efficiency, which means that this strategy has the potential to inaccurately estimate endogenous Ras abundance. The quantitation challenges associated with using this combined immuno-precipitation peptide standard approach can be seen with the independent observation of >100-fold lower concentration of Ras (20–150 pg/mg) in a panel of pancreatic and colorectal cancer samples [18].

To overcome issues that hinder accurate protein quantification using heavy peptides, we utilised protein standard absolute quantification. This quantification technique employs full-length Ras proteins, which addresses challenges associated with incomplete protein digestion, protein folding and peptide recovery to ensure that digestion conditions are identical between the protein standard and the endogenous protein [29]. We observed that SW48 cells harbour 90–220 ng of endogenous Ras per mg of total protein, equivalent to 260,000–540,000 Ras proteins per cell (Fig 4). Parental SW48 cells each harbour 65,000 copies of HRAS, 145,000 copies of KRAS4B and 82,000 copies of NRAS protein. We did not detect KRAS4A protein in SW48 cells; this is consistent with previous reports where only trace levels of KRAS4A mRNA were detected in this cell line [27]. Recent label free global quantification of protein abundance in HeLa cells revealed that the median protein copy number per cell was ~20,000 [17, 30]. This means that each of the Ras isoforms is of above average abundance, and together the Ras isoforms are in the top 15% of all proteins for relative abundance. Acute EGF stimulation results in up to 50% of cellular Ras proteins becoming activated [31] which corresponds to approximately 150,000 Ras molecules in SW40 cells. Whilst absolute quantitation of the other members of the Ras signalling cascade has not yet been conducted, label free quantitation estimates that there are ~5,000–60,000 copies per HeLa cell of the receptor tyrosine kinases (RTKs) EGFR and MET and isoforms of the Ras effectors Raf, MEK and ERK [17]. These data are required to parameterise RTK-MAP kinase network modelling and it will be interesting to determine the implications and consequences of the Ras signalling node being up to an order of magnitude more abundant than other local network nodes.

An important parameter for these models will also be the relative abundance of mutant KRAS proteins since dosage may be important for modulating oncogenesis and therapeutic responses [32]. The use of an isogenic panel of colorectal cancer cells differing only in the presence of a heterozygous KRAS mutation allowed the presence and influence of the most frequently observed tumour-associated variants to be characterised. Importantly, each variant is engineered to be expressed from the endogenous locus rather than as a result of ectopic over-expression. We detected mutant peptides in each of the isogenic cell lines providing a qualitative read-out suitable for biomarker screening (S4 Fig). Whilst our data represent an indirect measure of mutant KRAS abundance, we observed that a subset of KRAS mutant cell lines exhibited significantly increased expression of oncogenic KRAS (Fig 4D). Amplification of mutant versus wild type Ras alleles has been observed in human cancer cell lines and tumours and is associated with worse prognosis and resistance to RTK inhibitors [19, 32–36]. Each Ras codon mutation has a different transforming potential [37, 38], and it is tempting to speculate that the varied expression of mutant KRAS across the isogenic cell lines reflects adaptive titration of differentially potent oncogenic Ras variants.

The similar expression levels of the almost identical HRAS, KRAS and NRAS proteins suggests that no single isoform is likely to dominate signal outputs in wild type cells. The major location for Ras signalling is the plasma membrane where each Ras isoform resides within distinct signalling nano-clusters [39, 40]. Assuming an average SW48 cell diameter of 20 μm and approximately 50% of total Ras associated with the plasma membrane [23], we estimate that endogenous Ras is present at densities of ~100–210 molecules per μm^2^. Models of Ras nano-cluster size and function based on over-expression and immuno-labelling revealed a linear relationship between the number of nano-clusters and protein expression over several orders of magnitude [41, 42]. The endogenous densities that we estimate can now directly inform the modelling of Ras organisation and function with the cell surface.

Our data reveal that KRAS4B is the most abundant Ras isoform in a panel of SW48 cells; the 120,000–300,000 KRAS4B copies per cell typically represented 40–60% of total cellular Ras. These data are consistent with the observations that KRAS was the dominant isoform across a panel of cancer cells [15, 19]. The observation that KRAS is a highly expressed isoform is significant given the recent suggestion, based on *in vitro* experiments, that KRAS may be poorly expressed in comparison to the other Ras isoforms due to rare codon usage [5]. Together with the observation that Ras over-expression leads to senescence rather than tumourigenesis [43], Counter and colleagues suggest that low KRAS expression relative to HRAS and NRAS contributes to the particular oncogenic potency of KRAS. Consistent with this, conversion of KRAS rare codons to common codons increased KRAS protein expression and decreased *in vivo* tumourigenesis [44]. Whilst increased expression may promote senescence and reduce tumourigenesis, our data suggest that the preponderance of KRAS mutations in human cancers is not due to an oncogenically optimal low relative endogenous expression level of KRAS versus the other Ras isoforms.

In summary, we have optimised a robust method suitable for detecting mutant Ras and accurately quantifying endogenous Ras isoform protein abundance in sub-milligram quantities of cell lysates and likely extendable to tissue lysates. Amendments such as chromatography-based pre-fractionation may also open the approach up to clinical applications that would complement KRAS DNA sequencing-based screens currently used for determining treatment strategies. Finally, our measurements provide fundamental insights into Ras biology that are important for modelling Ras network behaviour and interpreting isoform-specific Ras signalling.

## Supporting Information

S1 Fig Concentration of the isotope-labelled (Lys8 Arg10), Ras PSAQ standards. (A) Colloidal blue staining of 1μg and (B) total light labelling of full-length, His-tagged KRAS4B-Ras. (C) Concentration of the light His-KRAS4B protein determined using independent methods: absorbance at 280 nm (A280) and bicinchoninic assay (BCA) that were performed in triplicate. The mass of the His-tag (17% of total protein) was deducted. (D) To accurately determine the concentration of heavy-labelled HRAS, KRAS4A, KRAS4B and NRAS proteins, a known amount of the accurately quantified light His-KRAS4B (250 or 500 ng) was mixed with volumes of isotope-labelled (Lys8 Arg10) PSAQ Ras standards and subject to in-gel tryptic digestion, before RP-HPLC and SRM analysis on a 4000 QTRAP, monitoring for light and heavy forms of the LVVVGAGGVGK peptide (transitions described in Supplementary S1 Table). Comparing the relative intensities of the different isotope signals enabled accurate measurement of the Ras PSAQ standards.
(EPS)

S2 Fig The extent of isotope labelling of the full-length, PSAQ Ras isoform standards. Each his-tagged, isotope-labelled (Lys8 Arg10) Ras protein standard was subject to separate in-gel digestions with trypsin and elastase, before analysis of light and heavy versions of LVVVGAGGVGK and FYTLVREI peptides using a 4000 QTRAP (AB SCIEX) in SRM mode. The standards were > 99% labelled on lysine residues, whilst no light version of the arginine containing peptide was detected.
(EPS)

S3 Fig Quantification of peptides shared between Ras isoforms. Example SRM traces for endogenous (Lys0 Arg0) and heavy (Lys8 Arg10) peptides describing H- and KRAS (TGEGFLCVFAINNTK) and K- and NRAS (SYGIPFIETSAK) cellular Ras abundance.
(EPS)

S4 Fig Mutant-specific Ras peptides. Mutant-specific Ras peptides were targeted using MRM-initiated detection and sequencing (MIDAS) from 1 μg of peptides derived from SW48 isogenic cell lines harbouring distinct, engineered KRAS mutations.
(EPS)

S5 Fig MS/MS spectra of all detected proteotypic Ras peptides in this study. Spectra obtained from Lys8 Arg10-labelled Ras peptides.
(EPS)

S1 Table Transitions for proteotypic Ras peptides. Transitions chosen for endogenous (Lys0 Arg0) and isotope-labelled (Lys8 Arg10) Ras peptides. Underlined residues indicate the presence of carbamidomethylation.
(EPS)
